# Synthesis, characterization and application of oligomeric proanthocyanidin-rich dual network hydrogels

**DOI:** 10.1038/s41598-023-42921-5

**Published:** 2023-10-18

**Authors:** Jie Song, Shuyu Zhang, Liuping Du, Chong Gao, Longyue Xie, Yu Shi, Ling Su, Yanli Ma, Shixue Ren

**Affiliations:** 1 Key Laboratory of Bio-Based Material Science & Technology (Northeast Forestry University), Ministry of Education, Harbin, 150040 People’s Republic of China; 2 College of Material Science and Engineering, Northeast Forestry University, Harbin, People’s Republic of China 150040; 3 College of Engineering and Technology, Northeast Forestry University, Harbin, People’s Republic of China 150040; 4 Yantai Vocational College, Yantai City, People’s Republic of China 264670

**Keywords:** Materials science, Bioinspired materials, Chemistry, Chemical synthesis

## Abstract

A structurally dense hydrogel, with strong hydrogen bonding networks, was formed from poly(vinyl alcohol), sodium alginate, and oligomeric proanthocyanidins, using a combination of freeze–thaw cycles and calcium ion cross-linking. The structure of the hydrogel was characterized by scanning electron microscopy and Fourier transform infrared spectroscopy. Mechanical testing and thermogravimetric analysis showed that incorporation of proanthocyanidins enhanced both the mechanical properties and the thermal stability of the hydrogel. The hydrogel was also demonstrated to have excellent ultraviolet resistance and antioxidant properties. The hydrogel was further shown that this hydrogel is also capable of generating electrochemical reactions, which strongly suggests that this hydrogel has exciting potential in many fields.

## Introduction

Hydrogels synthesized using different cross-linking methods have been widely used in the fields of biomedicine^1^, coatings^2^, sensors^3^ and energy-saving building materials^4^. The use of conductive hydrogels as electronic skins has attracted particular interest^5, 6^ and, because of their good film-forming and self-healing properties, it is feasible that hydrogels could be used as an "artificial skin"^5, 7^.

Interesting materials have been prepared from poly(vinyl alcohol) (PVA) and sodium alginate (SA). PVA has a variety of desirable properties, including good film-forming properties and degradability, hydrophilicity, resistance to abrasion, and elongation and tensile strength^8, 9^. SA, a naturally occurring polysaccharide consisting of -d-mannuronic acid (M) and -l-guluronic acid (G) units linked by (1 → 4) glycosidic bonds^10, 11^, is added to a wide range of foods as a gelling agent^12^. Blending PVA and SA produces a polymer solution that can be electrospun into nanofiber mats with good mechanical properties and thermal stability, probably because of the formation of hydrogen bonds^13^. A PVA/SA hydrogel, prepared using a green and simple process of freeze–thaw cycles, has been investigated as a slow release drug formulation^14^. The stability of PVA/SA hydrogels can be enhanced by introducing calcium ions, which cross-link to form a double network structure^15^. This dual network structure overcomes the poor mechanical properties of single network hydrogels and broadens the applications of hydrogels^16^. A PVA/SA hydrogel, formed by freeze-thawing and calcium ion cross-linking, had high water absorption and retention properties^17^ and cross-linked hydrogels, as well as being used as medical dressings, are desirable in the field of bioengineering^18, 19^. Despite good progress, existing hydrogels have obvious deficiencies in mechanical properties and thermal stability.

Tannins are plant secondary metabolites, consisting mainly of the flavonoid-3-ols (+)-catechin and (−)-epicatechin, which can be condensed to produce proanthocyanidins^20, 21^. Based on their molecular weight, proanthocyanidins are classified as monomers, oligomers and macromers^22^. Oligomeric proanthocyanidins (OPCs), which have good antioxidant and anti-inflammatory properties^22^, good biocompatibility^23^ and good UV resistance, and which are non-toxic and renewable, have found application in biomedical fields, such as the preparation of antibacterial materials^24^. OPCs are naturally occurring polyphenols and contain a large number of phenolic and aliphatic hydroxyl groups. Their highly branched structures facilitate the formation of hydrogen bonds with PVA/SA hydrogel systems^25, 26^, resulting in improved mechanical properties and thermal stability.

**Figure 1:**
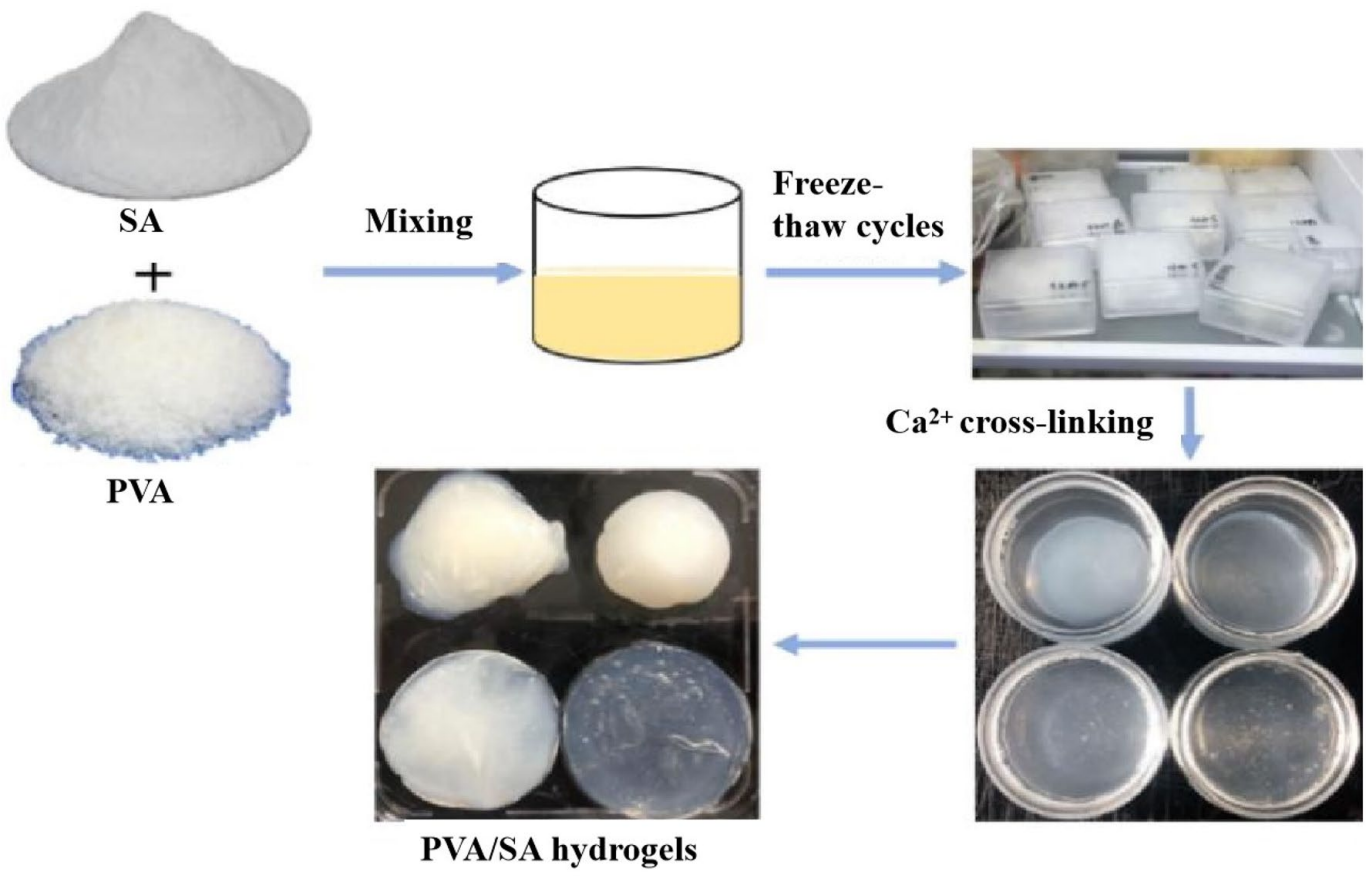
Flow chart of preparation of PVA/SA hydrogel.

**Figure 2:**
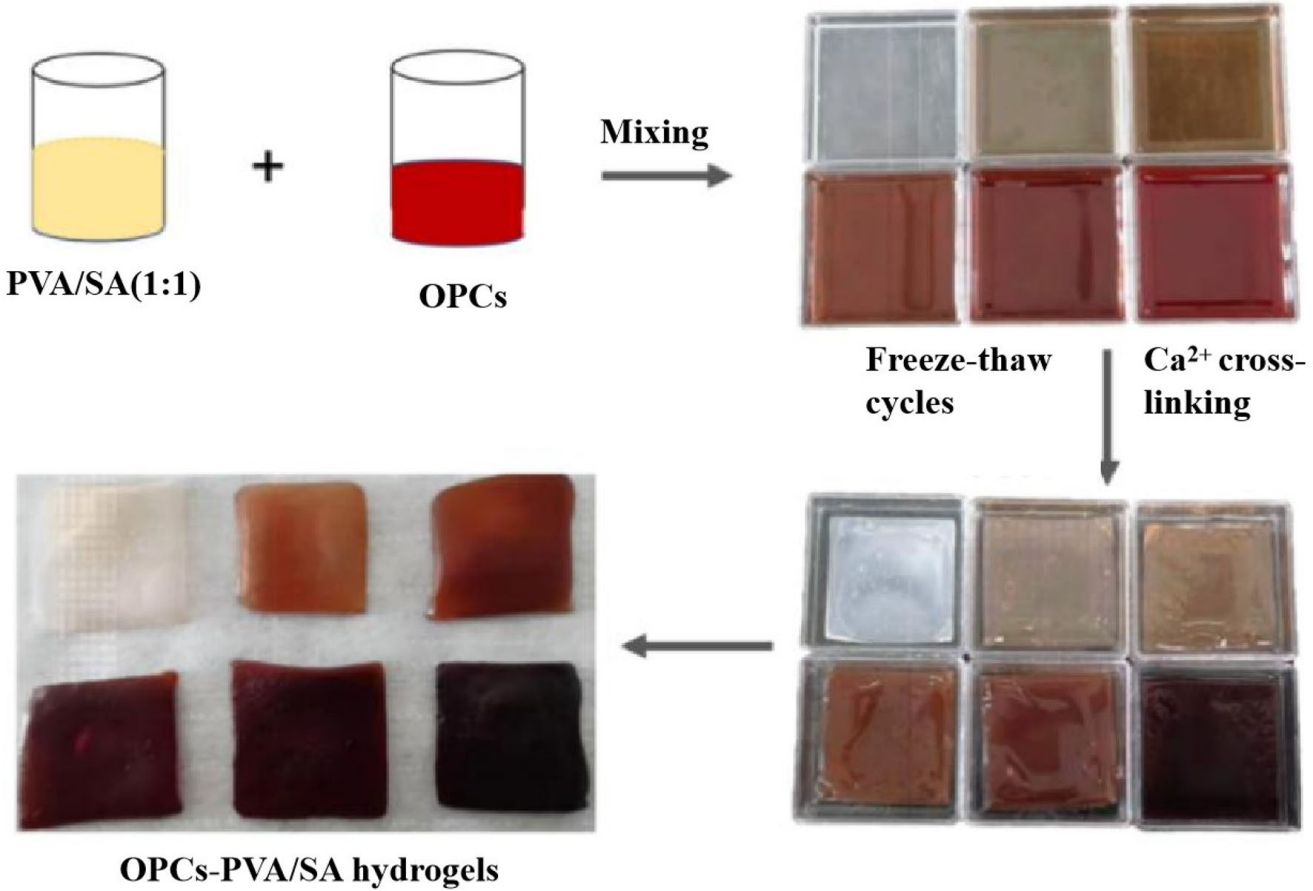
Flow chart of preparation of OPCs-PVA/SA hydrogel.

**Figure 3:**
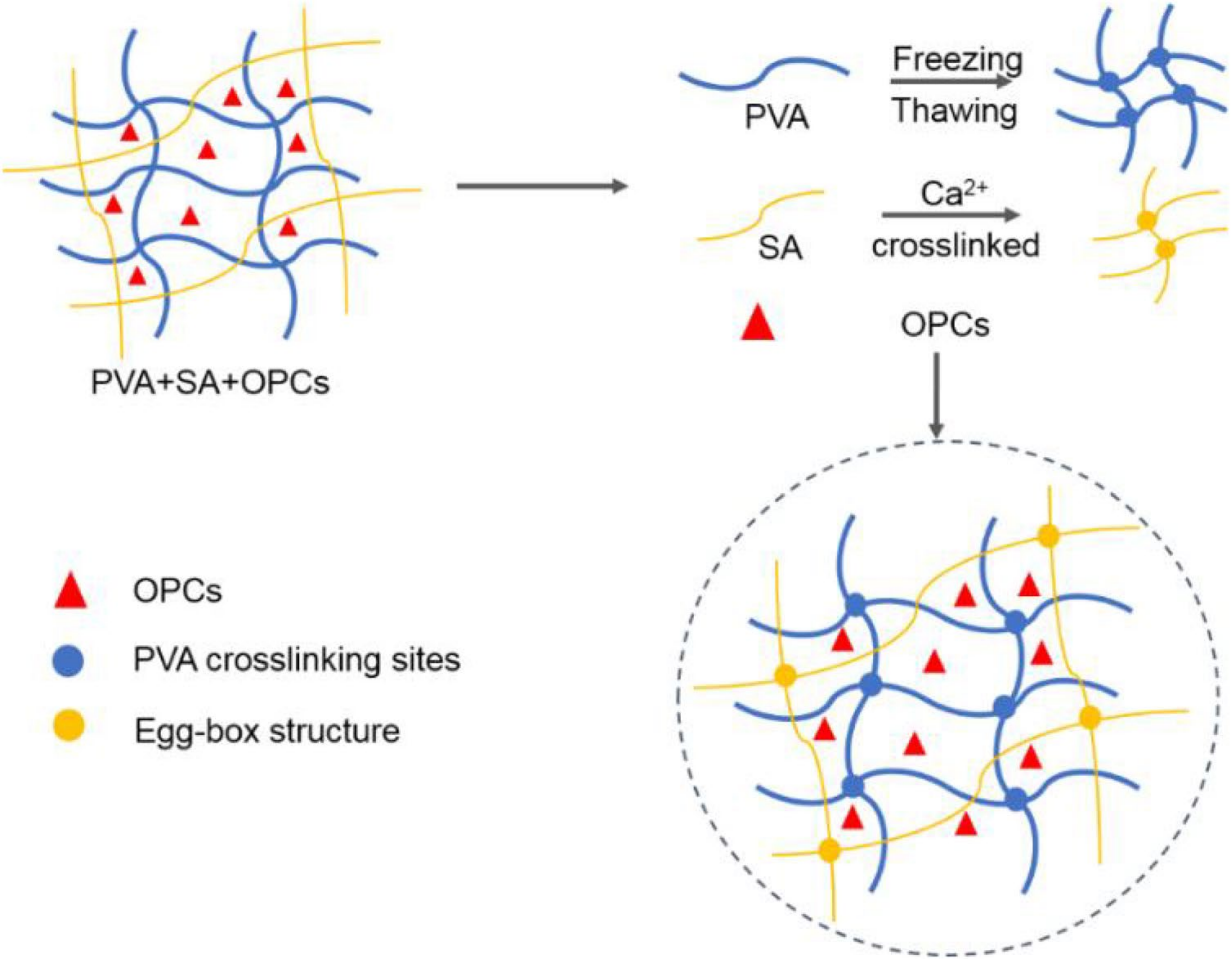
Schematic showing mechanism of formation of OPCs-PVA/SA hydrogel.

In this study, OPCs extracted from larch bark^27^ were used as fillers^28^, and OPCs-PVA/SA hydrogels were prepared using freeze–thaw cycles and calcium ion cross-linking (Figs. 1, 2). After repeated freeze–thaw cycles, the PVA molecular chains in the mixed solution become increasingly cross-linked by the formation of hydrogen bonds and eventually form a physically cross-linked network of PVA chains. This network was then cross-linked with a solution of calcium ions. The sodium ions of the G fragments of SA were rapidly replaced by calcium ions, forming an ‘egg-box’ structure, centered on the calcium ions. The OPCs become tightly locked in the hydrogel system where the two networks interpenetrate and interact with each other through hydrogen bonds, eventually forming a dense OPCs-PVA/SA hydrogel (Fig. 3). The formation of cross-linked networks was verified by scanning electron microscopy (SEM) and Fourier transform infrared (FTIR) analysis. The mechanical properties, thermal stability, UV stability, antioxidant properties and current induction properties of the hydrogels were also determined.

## Results

### Scanning electron microscopy

The PVA/SA hydrogels and The OPCs-PVA/SA hydrogels with different ratios were lyophilized using a freeze dryer. An electron scanning microscope (FEI QUATA200, FEI, The Netherlands) was used to observe the cross-sectional and surface microscopic morphology of the hydrogels.

**Figure 4:**
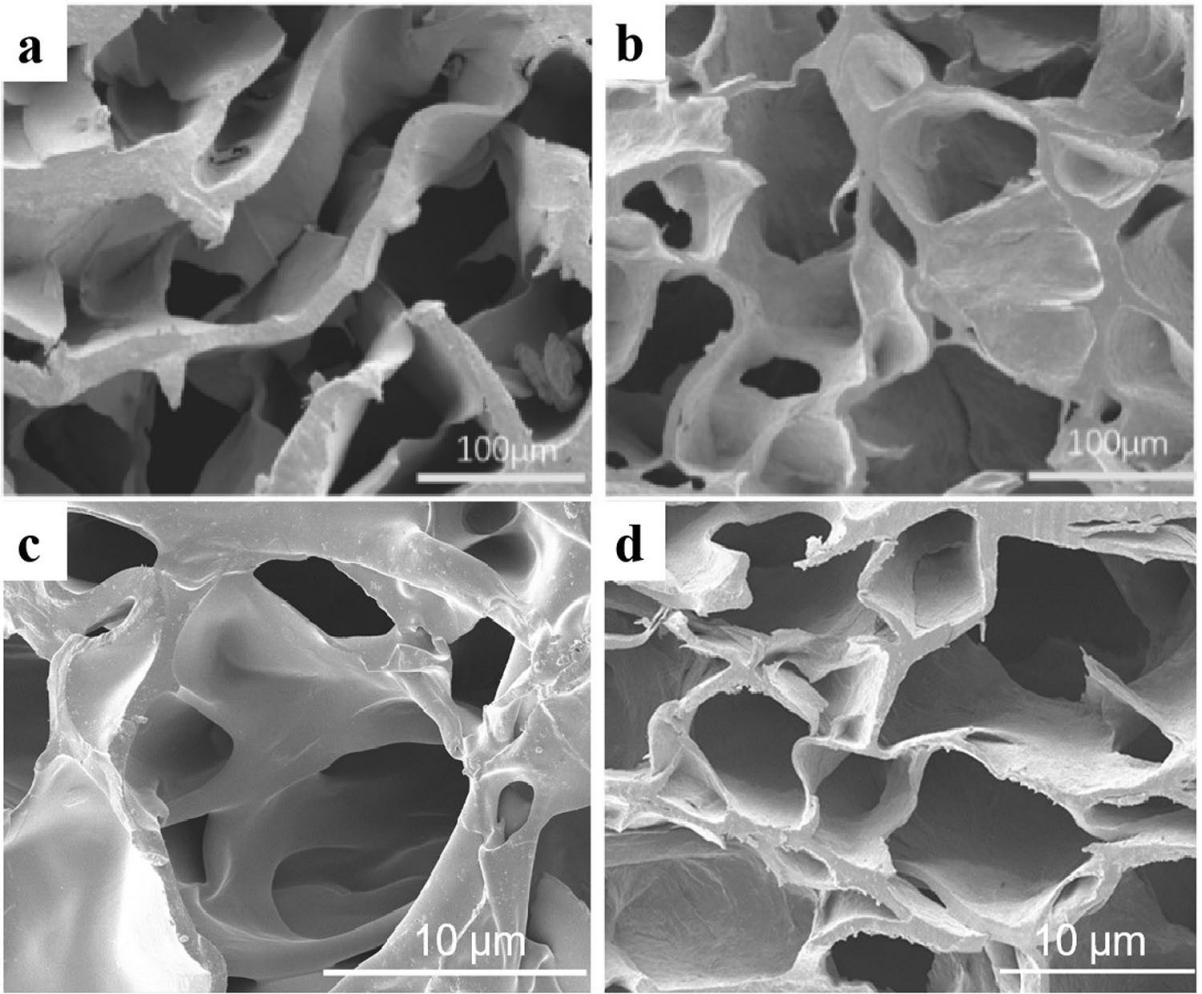
SEM image of SA hydrogel (**a**), SEM image of PVA/SA hydrogel (**b**), SEM image of OPCs-PVA/SA hydrogel (**c**, **d**).

SEM photomicrographs of the hydrogel containing 3% OPCs are shown in Fig. 4. It can be clearly seen that the internal morphology of the hydrogel is good, the pore sizes are uniform and the internal structure of the hydrogel is a three-dimensional spatial network. This indicates that the addition of OPCs did not disrupt the internal morphology of the PVA/SA hydrogel.

### Fourier transform infrared spectroscopy

The freeze-dried PVA/SA hydrogels and OPCs hydrogels with different mass concentrations (OPCs-PVA/SA hydrogels) were ground into powder. The IR spectra were obtained separately using a FTIR spectrometer (Perkin Elmer Inc., Viejo, MA, USA). The scanning range was from 4000 to 400 cm^−1^ with a resolution of 4 cm^−1^ and the samples were scanned 32 times.

**Figure 5:**
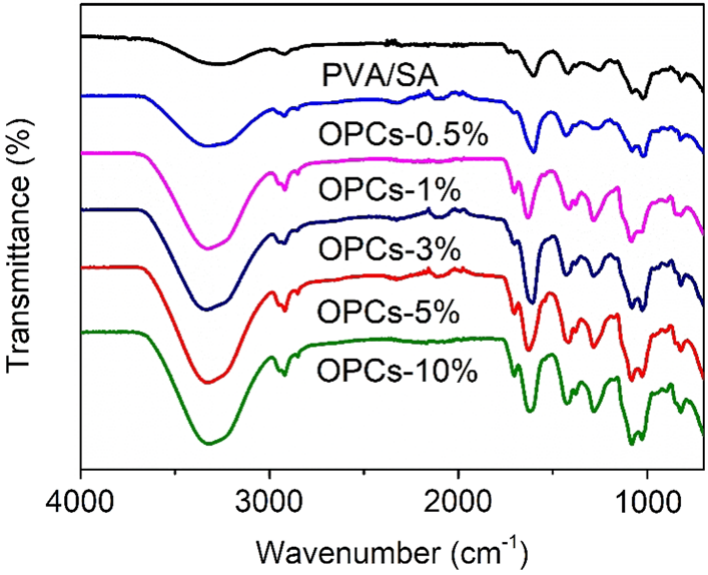
Infrared spectra of different PVA/SA hydrogel and OPCs-PVA/SA hydrogels.

FTIR spectra of PVA/SA and OPCs-PVA/SA hydrogels containing different amounts of OPCs are shown in Fig. 5. The broad absorption band present at ~ 3300−3600 cm^−1^ can be attributed to the stretching vibrations of inter- and intra-molecularly formed hydroxyl bonding (Formed by the interaction between alcohol hydroxyl groups on PVA and SA chains and phenolic hydroxyl groups on OPCs) within the gel. As the amount of OPCs in the PVA/SA gel increased, the absorption peak of the –OH groups shifted to higher wave numbers, with the peak moving from 3278 cm^−1^ (PVA/SA) to 3370 cm^−1^ (OPCs-10%). The intensity of the –OH stretching vibration was also significantly enhanced32. This indicates that the addition of OPCs increases the amount of aliphatic and phenolic hydroxyl groups in the gel, leading to the formation of new hydrogen bonds between the phenolic hydroxyl groups in the OPC molecules and the hydroxyl groups in the PVA/SA hydrogel and enhancing the interactions between them. This result can also provide a theoretical basis for the subsequent enhancement of mechanical properties and thermal stability. The out-of-plane stretching vibration of C–H groups appears at ~ 2931 cm^−1^. For comparison, the symmetric and asymmetric stretching vibrations of the –COO– (C=O) bonds of SA appear at 1631 cm^−1^ and 1425 cm^−1^ in the FTIR spectrum of the control (alginate film). The addition of OPCs to PVA/SA gels does not, however, cause significant changes in the amide (C=O) peaks, suggesting that these chemical groups are not involved in the strong polyphenol-biopolymer interactions.

### Differential scanning calorimetry

The DSC of different hydrogel samples was monitored simultaneously using a differential heat-thermogravimetric analyzer (NETZSCH Instrument Manufacturing GmbH, STA449F3, Germany). The test conditions were the same as for thermogravimetry: sample mass of 5 mg, flow control of 40 mL/min, temperature range of 20 °C–300 °C, and a ramping rate of 10 °C/min.

**Figure 6:**
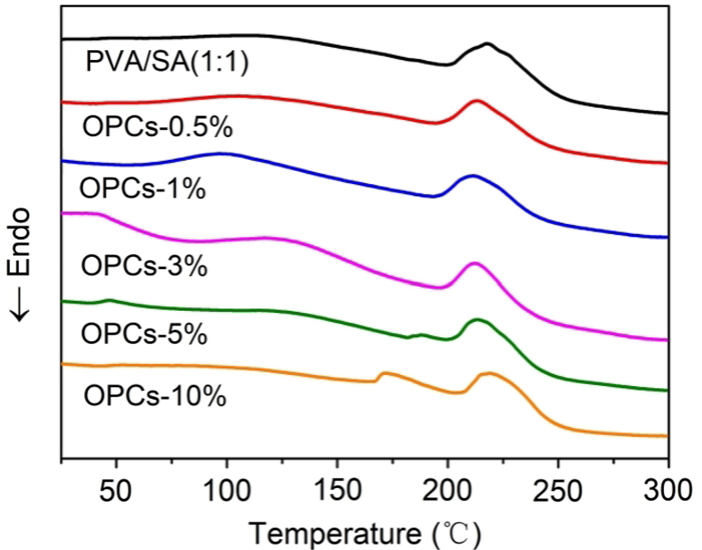
DSC curves of OPCs-PVA/SA hydrogels with different mass concentrations.

The glass transition temperature (Tg) of PVA/SA hydrogel without OPCs was 162 °C. The Tg of the OPCs-PVA/SA hydrogels increased from 157.9 to 188.9 °C as the mass concentration of OPCs was increased from 0.5 to 10% (Fig. 6). The addition of OPCs thus increased the Tg of the PVA/SA hydrogel, providing an explanation for the following study of mechanical properties.

### Thermogravimetric analysis

The thermal weight of OPCs-PVA/SA hydrogels was tested using a differential thermal-thermogravimetric analyzer (NETZSCH, STA449 F3). The test conditions were as follows: nitrogen was used as the protective gas, the mass of the hydrogel was about 5 mg, the flow rate was controlled at 40 mL/min, the temperature range was 20 °C–300 °C, and the temperature rise rate was 10 °C/min.

**Figure 7:**
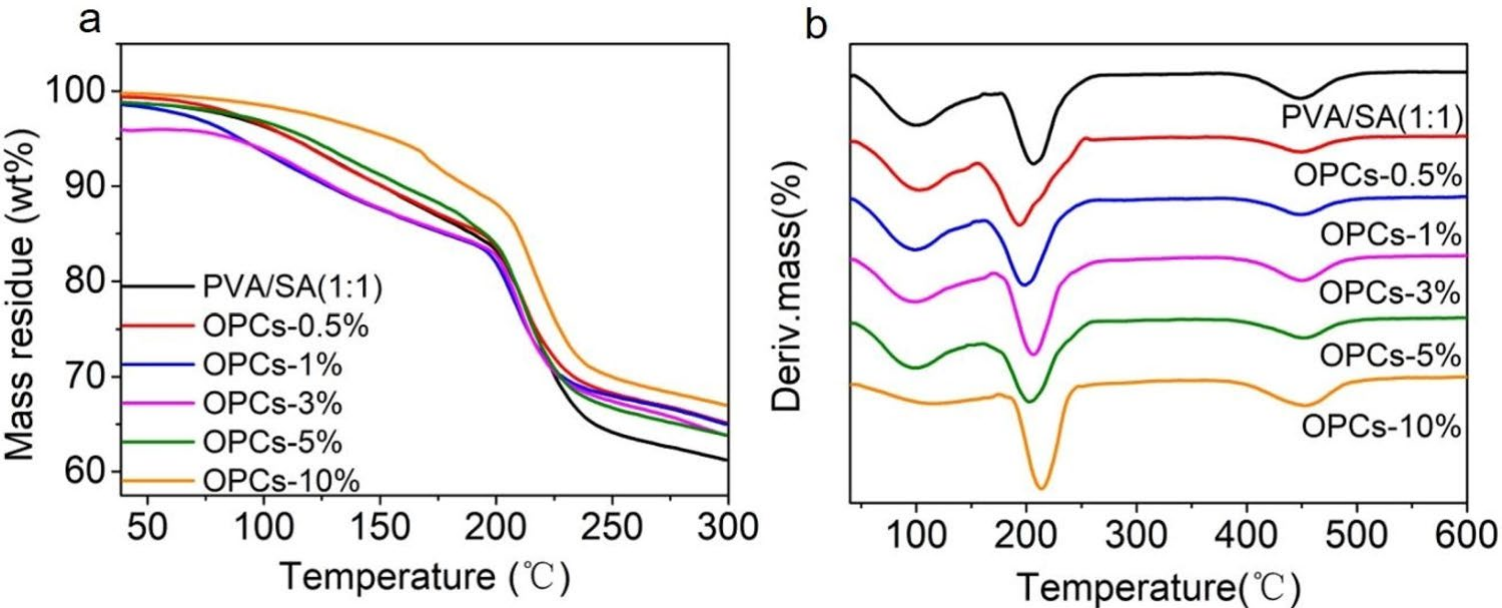
Infrared spectra of different PVA/SA hydrogel and OPCs-PVA/SA hydrogels. (**a**, **b**) show TG and DTG curves of OPCs-PVA/SA hydrogels with different mass concentrations, respectively.

Since thermal stability is an important property of hydrogels, the gel specimens were analyzed using a thermogravimetric analyzer. The thermal stabilities of OPCs-PVA/SA hydrogels containing different proportions of OPCs are shown in Fig. 7.

There is a significant difference between the thermogravimetric curves of the PVA/SA hydrogel and the different OPCs-PVA/SA hydrogels (Fig. 7a). The thermal stability of the PVA/SA hydrogel was the worst, and the thermal stability of the OPCs-PVA/SA hydrogels appeared to increase significantly with increased loading of OPCs over a certain range. The thermal stability of the OPCs-PVA/SA hydrogels was greatest when the mass concentration of OPCs in the gels was 10% (OPCs-10%). The rate of weight loss and initial decomposition temperature increased by 17.1% and 12.1 °C, respectively, compared with the PVA/SA hydrogel. The derivative thermogravimetric curves (Fig. 7b) show that the maximum decomposition temperature (Tmax) of the OPCs-10% hydrogel was increased by 7.1 °C compared with the PVA/SA hydrogel. The addition of OPCs thus improves the thermal stability of the PVA/SA hydrogel to some extent.

### Analysis of mechanical performance

The different hydrogels were tested in tensile and compression experiments using an electronic universal testing machine. Tensile performance results and stress-strain curves were obtained. Three sets of parallel samples were tested for each sample and the average value was calculated.

**Figure 8:**
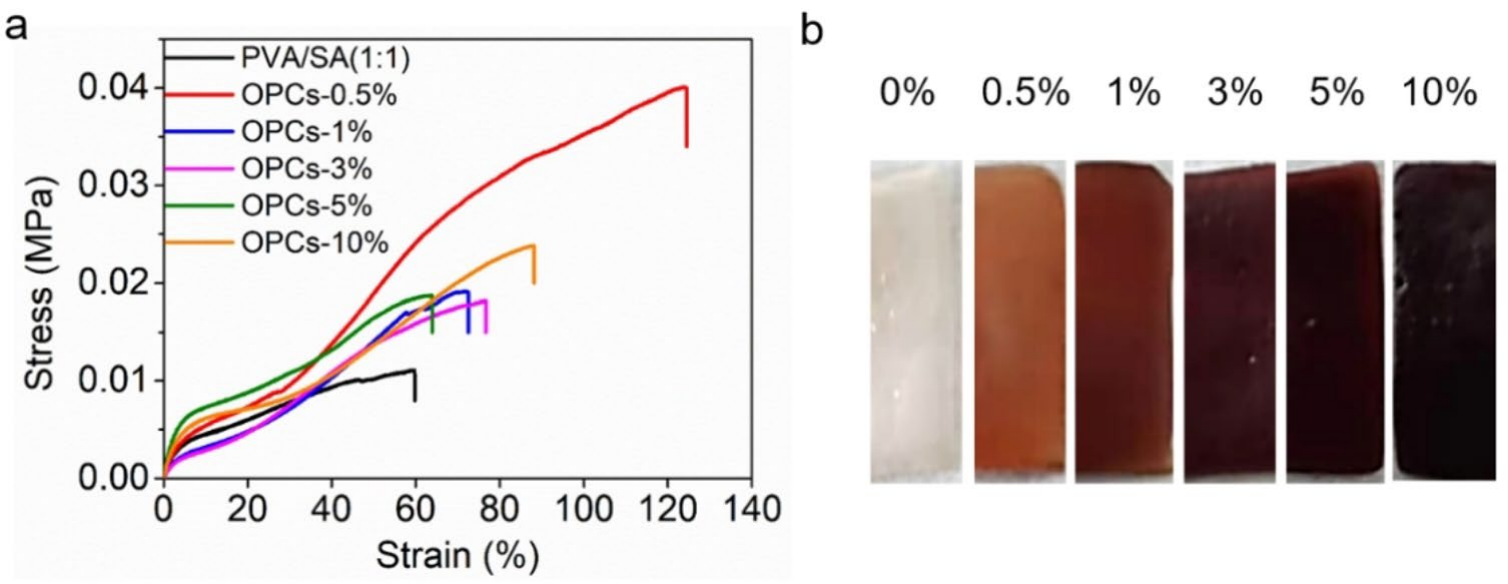
(**a**) Tensile stress–strain curves of different OPCs-PVA/SA hydrogels, (**b**) Macroscopic images of different OPCs-PVA/SA hydrogels.

The tensile properties (Table 1) and stress–strain curves (Figure 8a) show that the addition of different mass concentrations of OPCs improved the tensile strength of the hydrogels compared with PVA/SA hydrogel. The tensile strength of the OPCs-PVA/SA hydrogels reached a maximum (0.040 MPa) when the mass concentration of OPCs was 0.5% and then decreased as the mass concentration of OPCs was further increased. The tensile strength of the OPCs-PVA/SA hydrogels (0.018–0.040 MPa) was, however, always higher than that of the PVA/SA hydrogel (0.011 MPa). The addition of OPCs thus improved the tensile strength of the hydrogels to some extent. Macroscopic images of the different OPCs-PVA/SA hydrogels are shown in Fig. 8b.

**Table 1:** Tensile properties of different OPCs-PVA/SA hydrogels.

Concentration of OPCs (%)	0	0.5	1	3	5	10
Tensile strength (MPa)	0.011	0.040	0.019	0.018	0.020	0.024
Elongation at break (%)	117.95	131.67	138.46	151.26	139.51	166.83

Cylindrical samples of the hydrogels were tested in compression experiments using an electronic universal testing machine. The stress-strain curves of the hydrogels were recorded on the PC.

**Figure 9:**
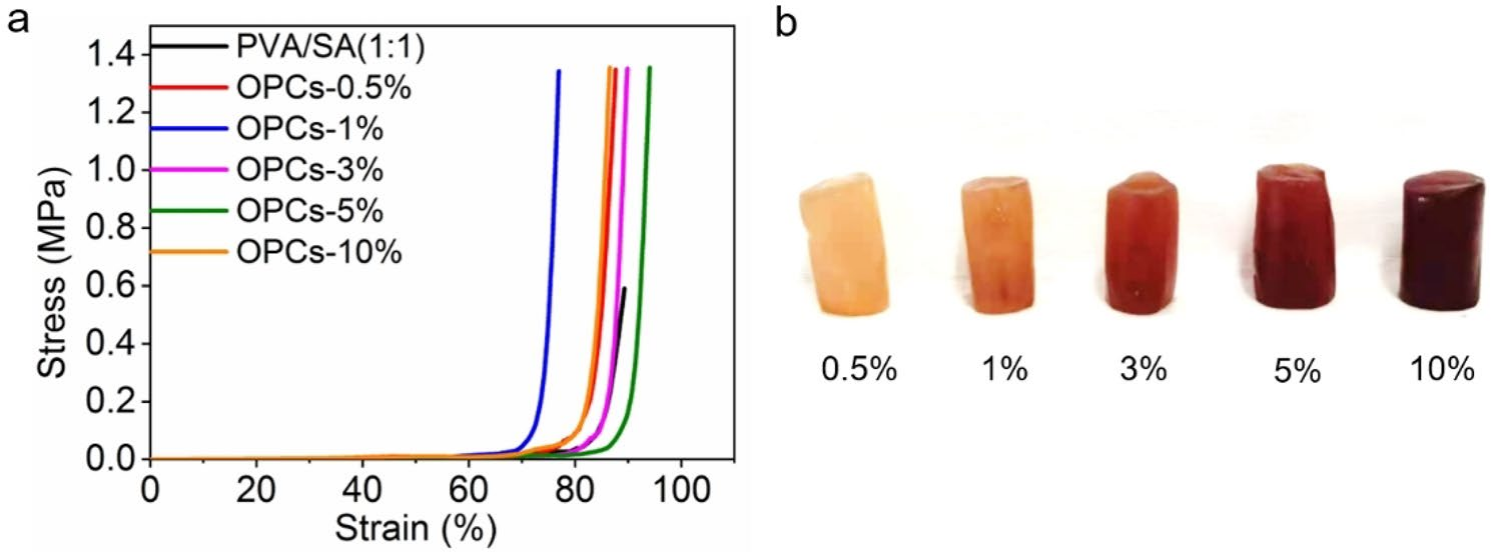
(**a**) Compressive stress–strain curves of different OPCs-PVA/SA hydrogels, (**b**) Macroscopic images of OPCs-PVA/SA hydrogels.

The compressive stress–strain curves of the hydrogels are presented in Fig. 9, and combining these with the analysis in Table 2, it can be concluded that addition of OPCs to the hydrogels increased their compressive resistance to some extent. Addition of moderate amounts of OPCs thus enhances the mechanical properties of the hydrogels.

**Table 2:** Compressive strength of different OPCs-PVA/SA hydrogel specimens.

Concentration of OPCs (%)	0	0.5	1	3	5	10
Compressive strength (MPa)	0.215	0.943	0.558	1.356	1.346	1.353

### Ultraviolet resistance

In the preparation of materials, UV resistance indicates the protection factor against UV. The lower the UV transmittance, the higher the absorption of UV and the higher the UV resistance of the material. The UV transmittance of OPCs-PVA/SA hydrogels with different mass concentrations was tested using a double-beam UV-Vis spectrophotometer (TU-1901 type, Beijing Pu-Analysis General Instrument Co., Ltd.). The gels were cut into 1 cm × 2 cm rectangles and clamped onto the fixed film apparatus of the UV spectrometer. Test conditions: Air was used as the background, the scanning range was 200 nm-800 nm, and the scanning step was 1 nm. Three sets of parallel samples were tested for each sample and the average value was calculated.

**Figure 10:**
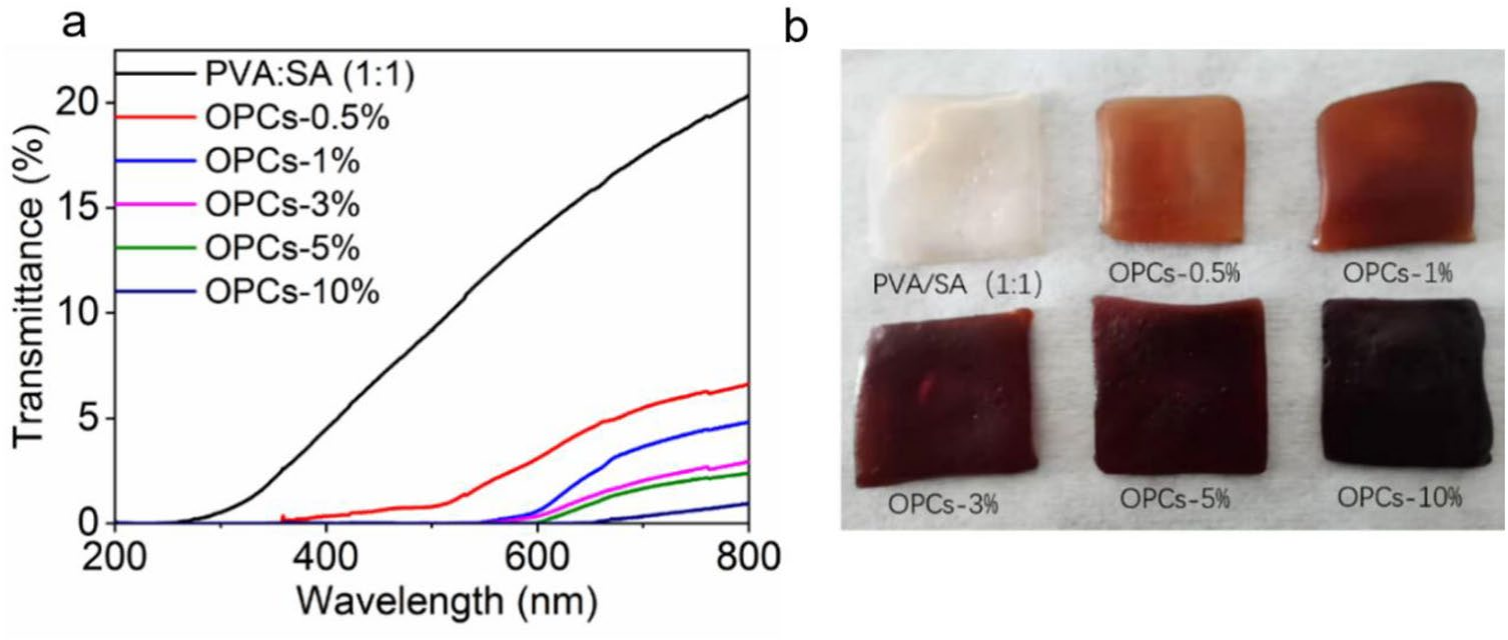
(**a**) Ultraviolet absorption spectra of OPCs-PVA/SA hydrogels of different concentrations, (**b**) Macroscopic diagram of OPCs-PVA/SA hydrogels.

The UV transmittance of the PVA/SA hydrogels gradually decreased as the mass concentration of OPCs increased (Fig. 10). When the concentration of OPCs reached 10% (OPCs-10%), light over the whole UV spectrum (200–400 nm) was completely blocked, with zero transmittance; transmittance in the visible range was also extremely low. The transmittance of light by the hydrogel is governed both by the color of hydrogel and the phenolic hydroxyl groups provided by the OPCs. When the mass concentration of OPCs in the hydrogel is low, the main factor affecting transmittance is absorption of UV light by the phenolic hydroxyl groups in the OPC molecules; when the mass concentration of OPCs is increased, the color of the hydrogel gradually deepens and transmittance by the hydrogel in the visible range gradually decreases. The addition of OPCs in certain amounts thus enhances the UV resistance of PVA/SA hydrogels.

### Antioxidant properties

The antioxidant properties of the hydrogels were assessed by measuring their ability to scavenge DPPH∙.Weigh 4 mg of DPPH and dissolve it in 100 mL of anhydrous ethanol (0.04 mg/mL) and store it away from light. OPCs of different mass concentrations were added to SA/PVA (1:1) hydrogel solution in turn and mixed well. Subsequently, it (0.7 mL) was added dropwise to DPPH solution (3 mL) and the reaction was kept away from light for 15 min. The absorbance of the solution at 517 nm was measured using a double-beam UV spectrophotometer with anhydrous ethanol as the control group. Three sets of parallel samples were tested for each sample and the average value was calculated.

**Figure 11:**
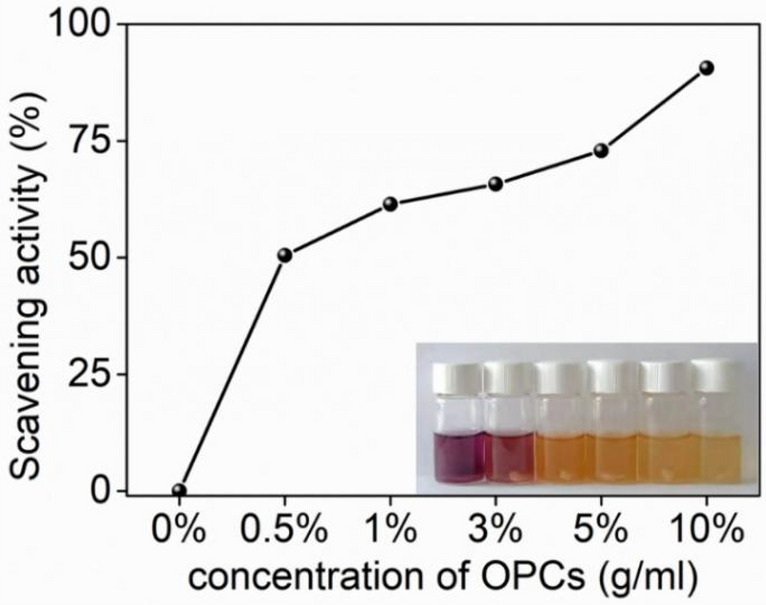
Antioxidant tests of OPCs-PVA/SA hydrogels with different mass concentrations.

OPCs are natural polyphenols with a large number of phenolic hydroxyl groups (o-phenolic hydroxyl groups) in their molecular structure, which have a strong role in scavenging free radicals and thus have good antioxidant properties. dPPH ethanol solution is dark purple with a maximum absorption peak at 517 nm, after adding the hydrogel sample with reducing ability to the dPPH ethanol solution, the color of the solution changes (fading of purple color) after a period of time and the absorbance at 517 nm decreases. The color of the solution changed (fading of purple color) and the absorbance at 517 nm decreased. The PVA/SA hydrogel without OPCs had no ability to scavenge DPPH∙ but the addition of increasing amounts of OPCs enhanced scavenging activity, as indicated by the fading of the color of the solution, With the increasing concentration of oligomeric proanthocyanidins in OPCs-PVA/SA hydrogels, the scavenging ability of DPPH∙ increased from 44.5 to 95.1%, accompanied by the fading of the solution color (Fig. 11). OPCs-PVA/SA hydrogels thus have strong antioxidant ability.

### Electrochemical response

**Figure 12:**
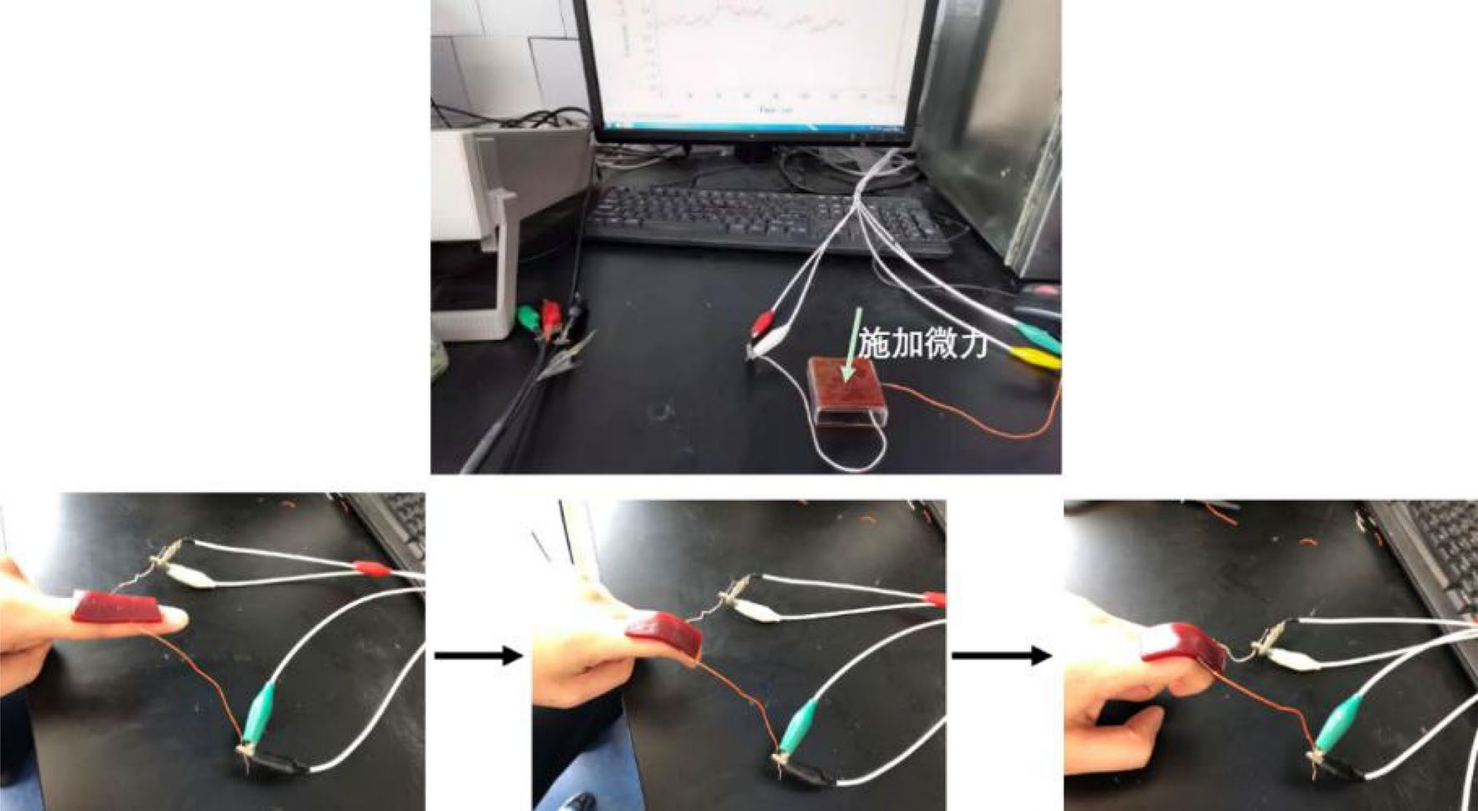
Schematic diagram of electrochemical testing.

**Figure 13:**
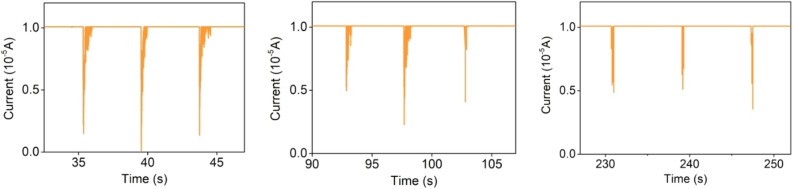
Time–current curves of OPCs-10% hydrogel.

The hydrogel was applied to a finger and changes in the current over time were measured on flexing the finger for the same period of time (Fig. 12). The time-current curves (Fig. 13) indicate that the current appears to have a regular response to the application of pressure and the degree of finger bending. The OPCs-PVA/SA hydrogels thus appear to have a certain sensing ability, suggesting that they could be used as an electronic skin.

## Discussion

A structurally dense OPCs-PVA/SA hydrogel, with extensive hydrogen bonding networks, was prepared by incorporating OPCs as fillers into PVA/SA hydrogels, using a combination of freeze–thaw cycles and calcium ion cross-linking. Scanning electron microscopy of the resulting hydrogels demonstrated that the addition of OPCs did not disrupt the internal morphology of the PVA/SA hydrogel, which retained its three-dimensional network structure. FTIR spectroscopy showed that the addition of OPCs led to an increase in the number of aliphatic and phenolic hydroxyl groups in the gels, resulting in the formation of new hydrogen bonds between the phenolic hydroxyl groups in the OPCs and the aliphatic hydroxyl groups in the PVA/SA hydrogel, with enhanced interactions between the two. There were no significant changes in the amide (C=O) peaks, indicating that these chemical groups were not involved in formation of the strong polyphenol-biopolymer interactions. The addition of OPCs in appropriate amounts improved the mechanical properties, thermal stability, UV resistance and antioxidant properties of the PVA/SA hydrogels. Electrochemical analysis showed that the OPCs-PVA/SA hydrogels have some sensing ability, strongly suggesting that they could be used as sensing materials in simulation and modeling applications. In summary, OPCs-PVA/SA hydrogels with good ultraviolet resistance, antioxidant properties and bioactivity have been prepared and have excellent potential for use as a sensing material in the field of electronic skin.

## Methods

### Materials

Analytical grade SA, anhydrous calcium chloride, analytical grade ethanol, analytical grade ether and analytical grade ethyl acetate were purchased from PLEASE ADD COMPANY NAME (Tianjin, China). PVA (degree of polymerization 1650–1850, degree of hydrolysis 86–90%) was purchased from PLEASE ADD COMPANY NAME (Qingdao, China). 2,2-Diphenyl-1-picrylhydrazyl was purchased from PLEASE ADD COMPANY NAME AND ADDRESS.

### Preparation of PVA/SA hydrogel

Accurately weighed a certain mass of PVA, dissolved in deionized water at 90 ℃, configured into an aqueous solution of polyvinyl alcohol with a mass concentration of 10% (according to the literature the gel formed by PVA at this concentration has better performance). Weigh the sodium alginate dissolved in deionized water at the same temperature, configured into an aqueous solution of sodium alginate with a mass concentration of 2%. Finally, the two solutions were configured into a mixed solution at a mass ratio of 1:1, which were frozen in a refrigerator at − 20 °C for 12 h and thawed at room temperature for 3 h, and the process was repeated five times. After that its immersion in calcium chloride solution with a mass concentration of 5% for 24 h was cross-linked and finally prepared to form different morphologies of PVA/SA hydrogels.

### Preparation of OPCs-PVA/SA hydrogel

Firstly, the PVA and SA solutions were mixed under the condition of mass ratio of 1:1 (5 parts each); secondly, the extracted oligomeric proanthocyanidins solid powder (0.005 g, 0.01 g, 0.03 g, 0.05 g, 0.1 g) were dissolved in trace amount of aqueous ethanol solution (70%, w/w) and added to a certain volume of PVA/SA hydrogel mixture solution in turn The mixture was stirred well to make it fully dissolved; subsequently, it was put into the freeze–thaw cycle in the refrigerator for 5 times. Finally, the OPCs hydrogel solutions with different mass concentrations were soaked in calcium chloride solution (5%, w/w) for 24 h to obtain OPCs-PVA/SA hydrogel. (The OPCs-PVA/SA hydrogels with different mass concentrations were noted as OPCs-0.5%, OPCs-1%, OPCs-3%, OPCs-5%, OPCs-10%).

## Data Availability

The datasets generated and/or analysed during the current study are not publicly available due [REASON WHY DATA ARE NOT PUBLIC] but are available from the corresponding author on reasonable request.
